# Disrespectful and abusive behavior during childbirth and maternity care in Ethiopia: a systematic review and meta-analysis

**DOI:** 10.1186/s13104-019-4118-2

**Published:** 2019-02-13

**Authors:** Zemenu Yohannes Kassa, Siraj Husen

**Affiliations:** 1 Department of Midwifery, College of Medicine and Health Sciences, Hawassa University, Hawassa, Ethiopia; 2 School of Medical Laboratory Science, College of Medicine and Health Sciences, Hawassa University, Hawassa, Ethiopia

**Keywords:** Meta-analysis, Childbirth, Maternity care, Disrespect, Abusive, Ethiopia

## Abstract

**Objective:**

Disrespectful and abusive behavior during childbirth and maternity care is violation of fundamental right of women and unborn child. There is scarce of data on disrespectful and abusive behavior during childbirth and maternity care in Ethiopia. The aim of this study was to determine disrespectful and abusive behavior during childbirth and maternity care in Ethiopia.

**Results:**

Seven studies were included in this meta-analysis of disrespectful and abusive behavior during childbirth and maternity care. The pooled prevalence of disrespect and abuse care during childbirth and maternity care was 49.4% (95% CI 30.9–68.1). Whereas physical abuse was 13.6% (95% CI 5.2–31.2), non-confidential care was 14.1% (95% CI 7.3–25.4), abandonment care was 16.4% (95% CI 14.7–18.2), and detention was 3.2% (95% CI 0.9–11.5). This study showed that disrespectful and abusive behavior during child birth and maternity care is high. Whereas, abandonment care is high. This study indicates that health care providers shall not leave women during childbirth and maternity care and listen women, federal minister of health and regional health bureau also identifying root of cause disrespect and abuse and to alleviate mistreatment during childbirth and maternity care.

**Electronic supplementary material:**

The online version of this article (10.1186/s13104-019-4118-2) contains supplementary material, which is available to authorized users.

## Introduction

Disrespectful and abusive behavior during child birth is a public health problem, which violates fundamental rights of women and unborn child. Disrespect and abuse treatment during childbirth in health facilities are a burning issue all over the world [1]. It is a common problem in maternal health care, and contributing to untold suffering and discouraging women for seeking care in health facilities. Women’s experiencing disrespect, abusive, or abandonment during child birth is an international agenda [2–4]. In the meantime, disrespect and abuse activities during child birth are physical abuse, non-consented care, non-confidential care, non-dignified care, discrimination based on specific attributes, abandonment or denial care and detention in the health facilities due to inability to pay [5, 6].

Pregnancy and childbirth are historic events that sustain offspring of families in every community across the globe. During pregnancy and childbirth woman’s positive and negative experience stay with her throughout her lifetime [7, 8]. Moreover, respectful maternity care (RMC) is a universal human right. RMC should routinely practice every woman irrespective her any background and in every health facilities around the world [9], and promoting respectful maternal care is vital components of the strategies to improve utilization and quality of maternity care during childbirth [10].

In Ethiopia has huge maternal and neonatal morbidity and mortality, concurrently low utilization of maternity care. The main to deterring pregnancy related complication and associated adverse pregnancy outcomes is ensuring regular and holistic care for all women throughout pregnancy and childbirth. And to increase maternity care utilization woman’s relationship with maternity care providers and the health facility system during pregnancy and childbirth is fundamental. Maternity care providers isn’t only the apparatus to utilize maternity care and potentially lifesaving maternity care services, while the feeling with maternity providers at this time have the impact to women’s memories of their childbearing experiences stay with them for a longtime and are often shared with other women and influences their decision to seek care from health facility [8, 11–13].

Every woman has the right to get quality of health care and with respect to her right during child birth and maternity care [14, 15]. There is limited data on disrespect and abuse during childbirth and maternity care in Ethiopia. This study is important to see the status of disrespect and abuse during childbirth and maternity care and it helps for maternity care providers, policy makers, federal minister of health, stakeholders, public health experts and clinician for possible alleviation of disrespect and abuse during childbirth and maternity care.

## Main text

### Method

#### Search strategy and quality appraisal

This systematic review and meta-analysis was carried out based on published studies. Articles were searched through an electronics on data bases: including, PubMed, Medline and HINARI, Google Scholar, Google and Cochrane Library were used to search articles. Articles were accessed by two reviewers (ZY and SH) using the following key terms, “attitude of health personnel” AND “delivery obstetrics*/nursing”, “maternity care” AND “disrespect”, “disrespect” OR “abuse”, “parturition” AND “prevalence”, “Professional Misconduct” AND “Professional-Patient Relations”, “disrespect “AND “Ethiopia”. For those studies having similar outcome of interest with the current objectives, their abstracts and the full-text were reviewed accordingly. The quality of each article was appraised by two independent reviewers (ZY and SH) using the Joana Brigg’s Institute (JBI) critical appraisal checklist for simple prevalence [16] using nine checklist tools. The criteria included the following tools: appropriate sample size, random selection of study sample, clear definition of the criteria for the inclusion of the study, use of objective criteria to assess the outcome of interest, reliable measurement of outcome variable, use of appropriate statistical analysis method. Assessment of articles using their title, abstract, and a full review of the articles were carried out before the inclusion of articles in the final meta-analysis. The discrepancies scoring during critical appraisal were resolved through discussion reviewing the articles by two authors. The quality assessment method was calculated by two reviewers’, which the articles scored greater than mean group as high quality score and the articles score less than mean as low quality score. Preferred Reporting Items for Systematic Reviews and Meta-Analyses (PRISMA) guidelines were strictly followed during the review and meta-analysis [17] (Additional file 1: Figure S1). Published articles in English were included. Searching of articles were conducted from January 01 to October 01, 2018.

#### Statistical analysis

Data entry and analysis were done using comprehensive meta-analysis (version 3.1). The summary of pooled prevalence of disrespect and abuse during childbirth with 95% CI was done using the random effects model, due to the possibility of heterogeneity among the studies.

#### Heterogeneity and publication bias

Heterogeneity and publication bias were assessed using I^2^ statistics and the Egger’s test respectively [18]. The heterogeneity of studies were tested using I^2^ test statistics. I^2^ test statistics result of 25%, 50%, and 75% was declared as low, moderate and high heterogeneity respectively. Statistical significant publication bias was declared at p-value less than 0.05 [19].

### Result

**Fig. 1:**
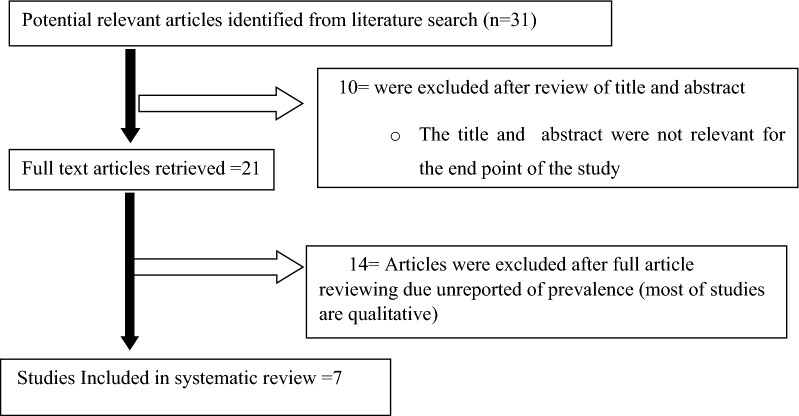
Flow diagram of the studies included in the meta-analysis

A total of 31 articles were identified through electronic data base search PubMed, Medline and HINARI, Google Scholar, Google and Cochrane Library. Articles were screened using their titles, abstracts and through full article review. Whereas 10 articles excluded due to duplication and 21 articles were reviewing full articles and 14 articles were excluded after full article reviewing due unreported of prevalence (most of studies are qualitative) (Additional file 2: Table S1). Finally, 7 studies were included in meta-analysis (Fig. 1). Heterogeneity test showed that I^2^ = 98.49%, p-value is 0.000 and publication bias (Egger’s test p-value is 0.21).

#### Operational definition

Disrespectful and abusive behavior during childbirth is any act of the following physical abuse, non-confidential care, non-consented care, non-dignified care, abandonment of care, discrimination and detention in the facilities.

#### Study characteristics

The total study population size involved in this systematic review and meta-analysis were 2493. Among these, 1535 were involved at community based studies, about 901 were laboring mothers and 57 were health care providers. The sample size of study population varied from 57 to 1125 (Table 1) [20–26].

**Table 1:** Prevalence of disrespect and abuse during childbirth and maternity care in Ethiopia: a systematic review and meta-analysis [20–26]

Author (refs)	Year of pub.	Country	Study design	Study population	Sample size	Over all pre. (%)	Physical abuse. pre (%)	Non confidential pre (%)	Detention pre. (%)	Abandonment. pre. (%)
Asefa and Bekele [20]	2015	Ethiopia	CS	Laboring mothers	173	78.6	32.9	21.4	0.6	39.3
Anteneh et al. [21]	2018	Ethiopia	CS	Providers	57	79.6	25.9	34.5	18	13.2
Sheferaw et al. [22]	2017	Ethiopia	CS	Laboring mothers	240	36.0	9.0	17.0	NR	19.0
Wasihun et al. [23]	2018	Ethiopia	CS	Community	410	67.1	57.6	11.0	NR	7.1
Wassihun and Zeleke [24]	2018	Ethiopia	CS	Laboring mothers	284	43.0	34.5	31.7	NR	32.4
Kathleen et al. [25]	2018	Ethiopia	CS	Laboring mothers	204	21.1	0.5	13.7	0	2.5
Gebremichael et al. [26]	2018	Ethiopia	CS	Community	1125	22.0	0.8	0.8	3.8	6.0

*H* HIV positive, *N* HIV negative, *P* health care providers, *C* community, *L* laboring mother, *CS* cross-sectional study

#### Meta-analysis

**Fig. 2:**
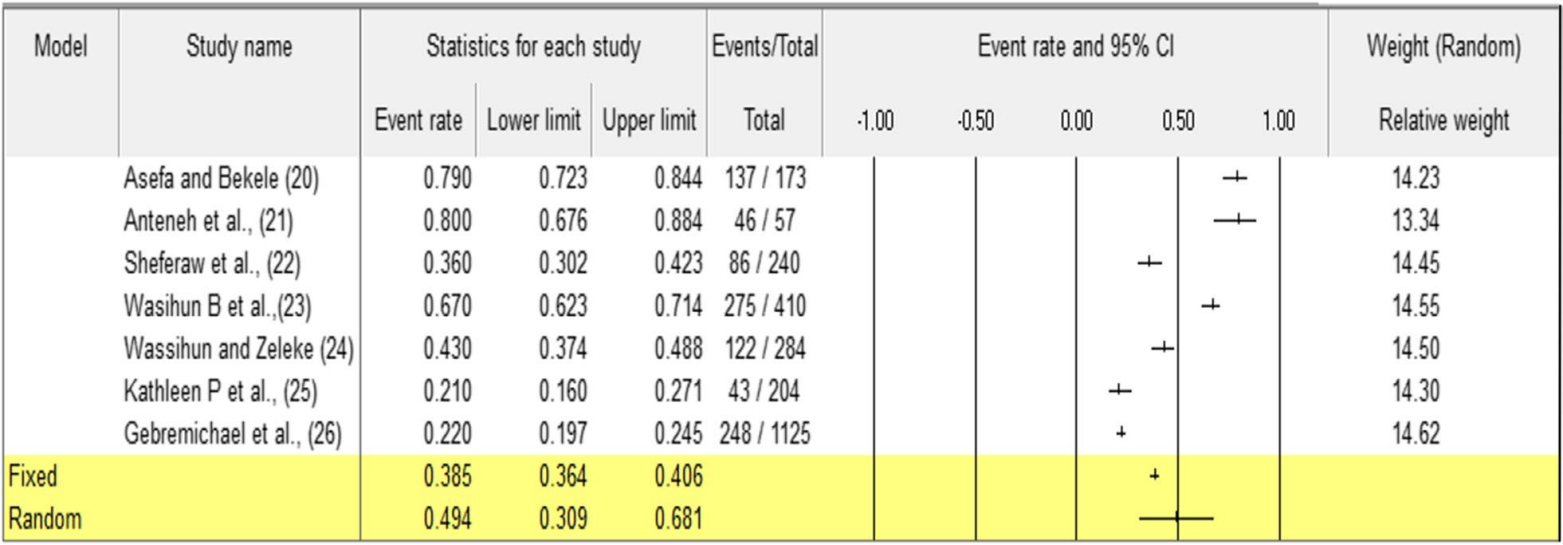
The forest plot Prevalence of disrespect and abuse during childbirth and maternity care in Ethiopia

The pooled prevalence of disrespectful and abusive behavior during childbirth and maternity care in Ethiopia was 49.4% (95% CI 30.9–68.1). The Cohran’s Q and I^2^ statistic for disrespect and abuse during childbirth and maternity care was 399.76 and 98.49% (Fig. 2). Pooled prevalence of physical abuse during childbirth and maternity care in Ethiopia was 13.6% (95% CI 5.2–31.2) (Additional file 1: Figure S1), pooled prevalence of non-confidence care during childbirth and maternity care in Ethiopia was 14.1% (95% CI: 7.3–25.4) (Additional file 1: Figure S2), pooled prevalence of abandonment during childbirth and maternity care in Ethiopia was 16.4% (95% CI 14.7–18.2), and pooled prevalence of detention during childbirth and maternity care in Ethiopia from 4 studies was 3.2% (95% CI 0.9–11.5) (Additional file 1: Figure S3).

### Discussion

Disrespectful and abusive behavior during childbirth and maternity care are violation of fundamental rights of woman and unborn child. It is a common problem in health facilities. In fact, promoting respectful maternity care during childbirth a vehicle to utilize maternal health care and preventing maternal and child morbidity and mortality by augmenting woman center care.

Disrespectful and abusive behavior during childbirth and maternity care have been documented in both high and low income countries, making this a truly a global agenda. Its operational definition is not straightforward and having a difference an intentional community. Bowsers and Hill’s in 2010 revealed that the evidence of disrespectful and abusive behavior during child birth and maternity care at facilities. They categorized into seven; physical abuse, non-confidential care, non-consented care, non-dignified care, abandonment of care, discrimination and detention in the facilities [2, 14, 27].

The aim of this systemic review and meta-analysis was to assess disrespectful and abusive behavior during childbirth and maternity care in Ethiopia. Seven studies were included in this systemic review and meta-analysis. The pooled prevalence of disrespectful and abusive behavior during child birth and maternity care was 49.4% in Ethiopia. This finding is higher than the study was done in India 28% [28], in Brazil 18.3% [29], in Mexico 37.7% [30]. The diffidence might be the individual difference of health care providers, the health facilities difference and health system difference and plus to that socioeconomic, cultural aspect, study time, data collection time, sampling technique and the way of defining of disrespect and abuse care in the studies.

This finding is lower than study done in Pakistan 97.4% [31], in Pakistan 99.7% [32], and in Peru 97.4% [33]. The diffidence might be the individual difference of health care providers, the health facilities difference and health system difference and plus to that socioeconomic, cultural aspect, study time, data collection time, sampling technique and the way of defining of disrespect and abuse care in the studies.

In this meta-analysis the highest prevalence was abandonment care during childbirth and maternity care 16.4%, whereas the lowest prevalence was detention during childbirth and maternity care 3.2%.

Implication of this study; produce pooled prevalence of disrespectful and abusive behavior during child birth and maternity care in Ethiopia. Decision makers, policy planners and clinician on maternal and child health to achieve sustainable development goal three to ensure health lives and promote wellbeing for all ages and the women has the right to get maximum standard of care during maternity care. Therefore, this meta-analysis is an input for policy planners for evidence-based strategy to alleviate disrespect and abuse during childbirth and maternity care at facilities.

### Conclusion

This study showed that disrespectful and abusive behavior during child birth and maternity care is high. Whereas abandonment care is high. This study indicates that health care providers shall not leave women during childbirth and maternity care and listen women, federal minister of health and regional health bureau also identifying root of cause disrespect and abuse and to alleviate mistreatment during childbirth and maternity care.

## Limitation

Potential limitations of this study, due to the nature of disrespectful and abusive behavior during childbirth and maternity care. Another important limitation is that different definition of disrespect and abusive behavior during childbirth and maternity care. The current estimates are limited to childbirth and maternity care. Disrespectful and abusive behavior can be occurred at family planning and another medical care. An important limitation is the use of childbirth and maternity care as search term.

## Additional files

**Additional file 1: Figure S1.** The forest plot Prevalence of physical abuse during childbirth and maternity care in Ethiopia. **Figure S2.** The forest plot Prevalence of non-confidential care during childbirth and maternity care in Ethiopia. **Figure S3.** The forest plot Prevalence of detention during childbirth and maternity care in Ethiopia.

**Additional file 2: Table S1.** PRISMA 2009 Checklist for disrespectful and abusive behaviors during childbirth and maternity care in Ethiopia.

## Abbreviations

WHO: World Health Organization
RMC: respectful maternity care
