# Supplementary material for: Disrespectful and abusive behavior during childbirth and maternity care in Ethiopia: a systematic review and meta-analysis
Source: BMC Res Notes. 2019 Feb 13;12:83. doi: 10.1186/s13104-019-4118-2 (PMC6375170; doi:10.1186/s13104-019-4118-2)
Supplement: Supplementary file 1 — Additional file 1: Figure S1. The forest plot Prevalence of physical abuse during childbirth and maternity care in Ethiopia. Figure S2. The forest plot Prevalence of non-confidential care during childbirth and maternity care in Ethiopia. Figure S3. The forest plot Prevalence of detention during childbirth and maternity care in Ethiopia. [file 13104_2019_4118_MOESM1_ESM.docx]

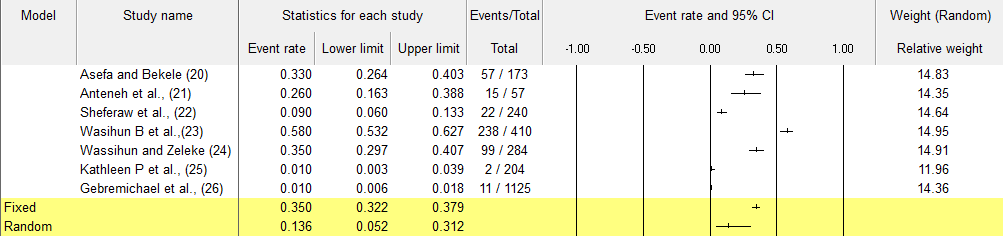


Fig. S1: The forest plot Prevalence of physical abuse during childbirth and maternity care in Ethiopia


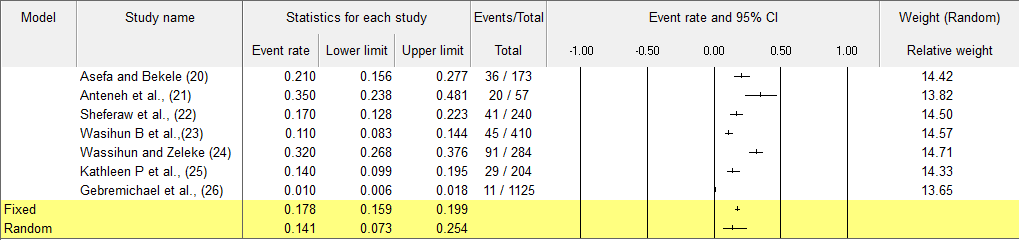


Fig. S2: The forest plot Prevalence of non-confidential care during childbirth and maternity care in Ethiopia


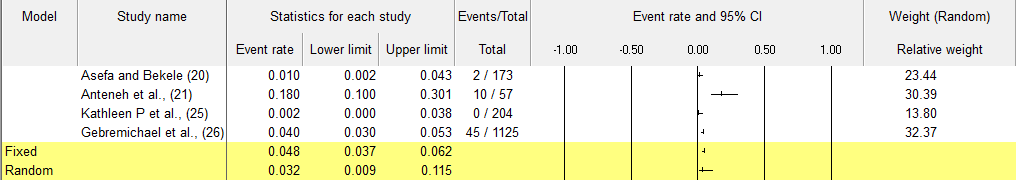


Fig. S3: The forest plot Prevalence of detention during childbirth and maternity care in Ethiopia
